# Case Report: Living on the Edge—Transcatheter Mitral Valve Repair Related Infective Endocarditis

**DOI:** 10.3389/fcvm.2021.810054

**Published:** 2022-01-06

**Authors:** Nicole Lewandowski, Ehssan Berenjkoub, Eduard Gorr, Marc Horlitz, Peter Boekstegers, Mirko Doss, Sami Sirat, Dennis Rottländer

**Affiliations:** ^1^Department of Cardiology, Krankenhaus Porz am Rhein, Cologne, Germany; ^2^Faculty of Health, Department of Cardiology, School of Medicine, University Witten/Herdecke, Witten, Germany; ^3^Department of Cardiology, Helios Klinikum Siegburg, Siegburg, Germany; ^4^Department of Cardiothoracic Surgery, Helios Klinikum Siegburg, Siegburg, Germany

**Keywords:** sepsis, endocarditis, edge-to-edge repair, mitral valve replacement, *Proteus mirabilis*

## Abstract

**Background:** Infective endocarditis (IE) following mitral valve edge-to-edge repair is a rare complication with high mortality.

**Case summary:** A 91-year-old male patient was admitted to intensive care unit with sepsis due to urinary tract infection after insertion of a urinary catheter by the outpatient urologist. Two weeks ago, the patient was discharged from hospital after successful transcatheter edge-to-edge mitral valve repair (TEER) using a PASCAL Ace device. The initially withdrawn blood revealed repeatedly *Proteus mirabilis* bacteremia as causal for the sepsis due to urinary tract infection. An antibiotic regime with Ampicillin/Sulbactam was initiated and discontinued after 7 days. During the clinical course the patient again developed fever and blood cultures again revealed *P. mirabilis*. In transesophageal echocardiography (TOE), IE of the PASCAL Ace device was confirmed by a vegetation accompanied by a mild to moderate mitral regurgitation. While the patient was stable at this time and deemed not suitable for cardiac surgery, the endocarditis team made a decision toward a prolonged 6-week antibiotic regime with an antibiotic combination of Ampicillin 2 g qds and Ciprofloxacin 750 mg td. Due to posterior leaflet perforation severe mitral regurgitation developed while PASCAL Ace vegetations were significantly reduced by the antibiotic therapy. Therefore, the patient underwent successful endoscopic mitral valve replacement. Another 4 weeks of antibiotic treatment with Ampicillin 2 g qds followed before the patient was discharged.

**Discussion:** *P. mirabilis* is able to form biofilms, resulting in a high risk for endocarditis following transcatheter mitral valve repair especially when device endothelization is incomplete. Endoscopic mitral valve replacement could serve as a bailout strategy in refractory Clip-endocarditis.

## Introduction

Infective endocarditis following transcatheter edge-to-edge mitral valve repair (TEER) is rare and occurs most often in the first months after device implantation, when endothelization is incomplete. *Proteus mirabilis* has a high ability of building biofilms and bacteremia, which poses a risk for colonization of cardiac devices (1). Therefore, patients with *P. mirabilis* bacteremia following TEER should be carefully monitored for infective endocarditis.

## Case Presentation

A 91-year-old male patient was admitted to the intensive care unit with fever (40.1°C), tachycardia (120 bpm), oliguria (30 ml/h) and hypotension (80/50 mmHg). Two weeks ago, the patient was discharged from hospital after successful transcatheter edge-to-edge mitral valve repair (TEER) using a PASCAL Ace device (Edwards Lifesciences, Irvine, CA, USA). TEER was scheduled by the local heart team to treat a severe functional mitral valve regurgitation due to heart failure with midrange reduced ejection fraction (HFmrEF). After implantation of a central (A2/P2) PASCAL Ace device, the mitral regurgitation could be reduced to a minimal residual jet. The post-interventional course was uneventful and the patient was discharged from hospital with negative inflammatory markers (leucocytes, CRP) and improved exercise capacity. The day before re-admission to hospital the patient faced urinary retention due to a known prostate hyperplasia, which was treated with a urinary catheter by the outpatient urologist.

**Figure 1:**
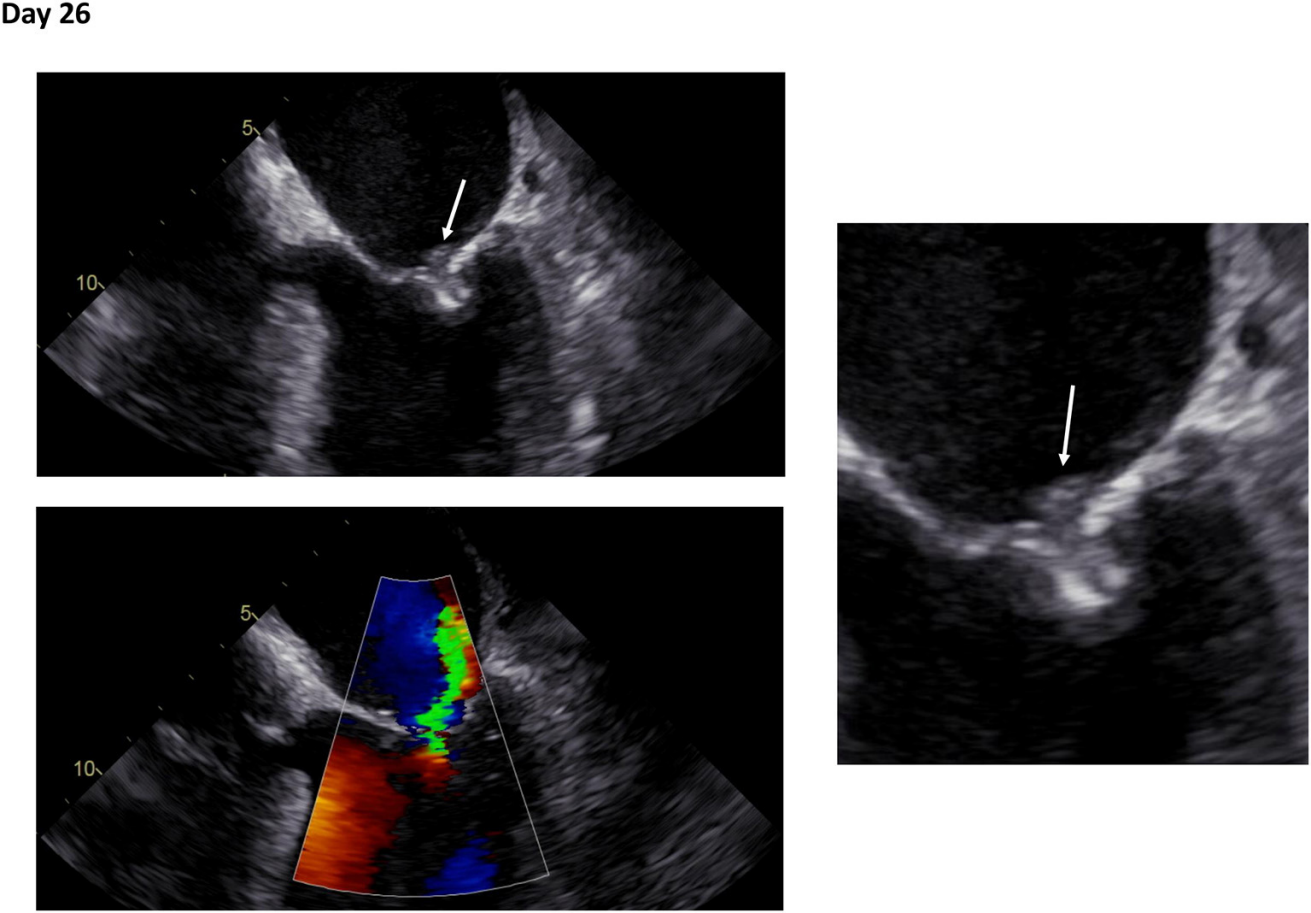
Initial transesophageal echocardiography in recurrent fever and persisting bacteremia. Transesophageal echocardiography at day 26 following mitral valve edge-to-edge repair. Right: Zoom on mitral valve. White arrows indicate an echogenic structure suspicious for vegetation (Day 26: Supplementary Videos 1, 2).

Vasopressors, infusion therapy and fever reduction led to hemodynamic stabilization of the patient. Pneumonia was excluded by a thoracic X-ray and abdominal sonography excluded post-renal kidney injury or an abdominal focus of sepsis. The laboratory results revealed a normal CRP (<0.4 mg/dl) but markedly elevated PCT (63.34 ng/ml), leucocytosis and acute to chronic renal failure, which led to the diagnosis of a sepsis due to urinary tract infection. Early antibiotic treatment with piperacillin/tazobactam 4.5 g tds was started immediately. The urine status proofed a bacterial urinary tract infection. On day 2 catecholamines could be reduced and PCT decreased (41.1 ng/ml), while CRP (18.6 mg/dl) was markedly increased. The initially withdrawn blood revealed repeatedly *P. mirabilis* bacteremia as causal for the sepsis due to urinary tract infection. Urine culture also confirmed *P. mirabilis*. The antibiograms of blood and urine culture revealed Ampicillin/Sulbactam, Amoxicillin/Clavulanate, Piperacillin/Tazobactam, Cefuroxim, Ceftriaxon, and Meropenem to be effective antibiotic agents. Following the antibiogram the antibiotic regime was changed to Ampicillin/Sulbactam 3 g tds. After reversal of renal failure and discontinuation of the vasopressors the patient was transferred to the general internal medicine ward. Inflammatory markers decreased. Transesophageal echocardiography (TOE) was performed to exclude infective endocarditis (IE). However, initial TOE interpretation excluded IE, while a small echogenic structure on the PASCAL Ace device was suspicious for vegetation (Figure 1; Supplementary Videos 1, 2). The antibiotics were discontinued, but the patient suffered from decompensated heart failure with peripheral edema and pleural effusion as consequence of the sepsis infusion therapy. Right side pleurocentesis was necessary. At day 35 the patient developed fever and blood cultures again showed *P. mirabilis* with the same resistogram. CRP and PCT were elevated but no signs of bacteriuria were found in the urine status. Clinical course of recurrent bacteremia with implanted cardiac device in mitral position led to the suspected diagnosis of endocarditis.

**Figure 2:**
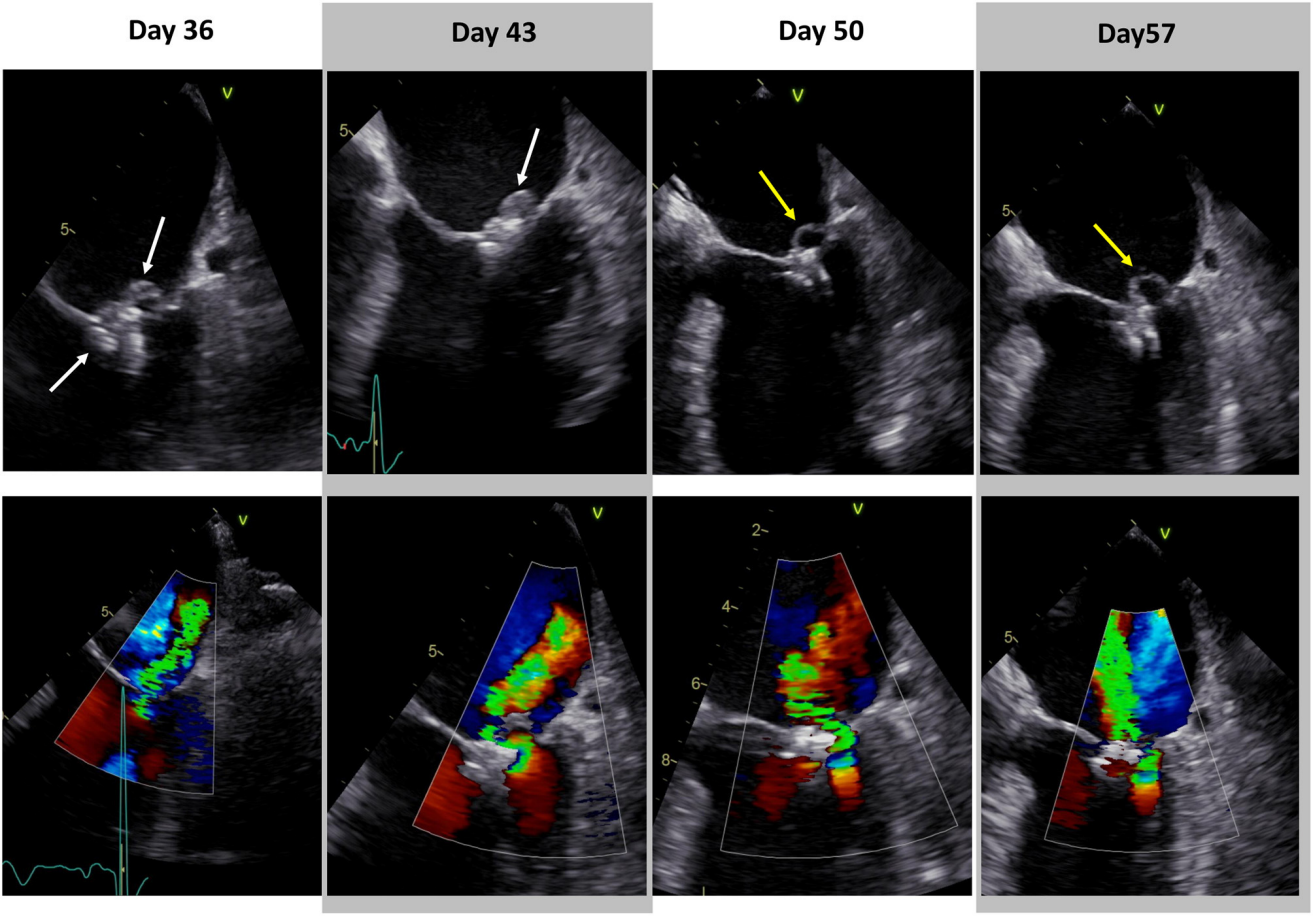
Progress of infective endocarditis in transesophageal echocardiography. Transesophageal echocardiography of infective endocarditis at various timepoints during calculated antibiotic therapy. Day 0 = transcatheter mitral valve edge-to-edge repair. White arrows indicate vegetations. Yellow arrows show perforated posterior mitral leaflet and significantly reduced vegetation (Day 36: Supplementary Videos 3, 4; Day 43: Supplementary Videos 5, 6; Day 50: Supplementary Videos 7, 8; Day 57: Supplementary Videos 9, 10).

**Figure 3:**
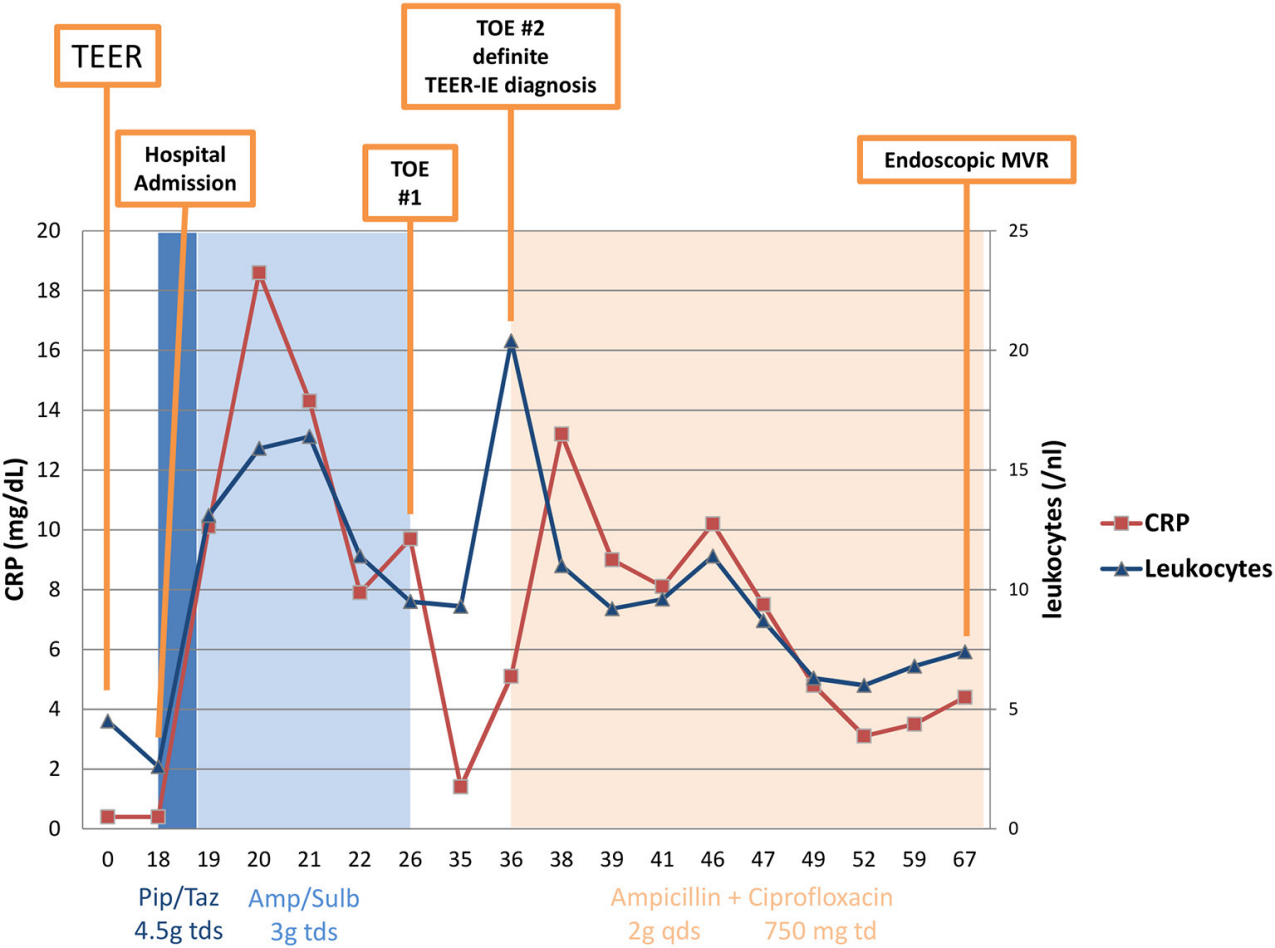
C-reactive protein and leucocytes over the clinical course of infective endocarditis. TEER, transcatheter edge-to-edge repair; TOE, transesophageal echocardiography; IE, infective endocarditis; MVR, mitral valve repair; Pip/Taz, piperacillin/tazobactam; Amp/Sulb, ampicillin/sulbactam; tds, three times a day; qds, four times a day; td, twice daily; CRP, C-reactive protein.

**Figure 4:**
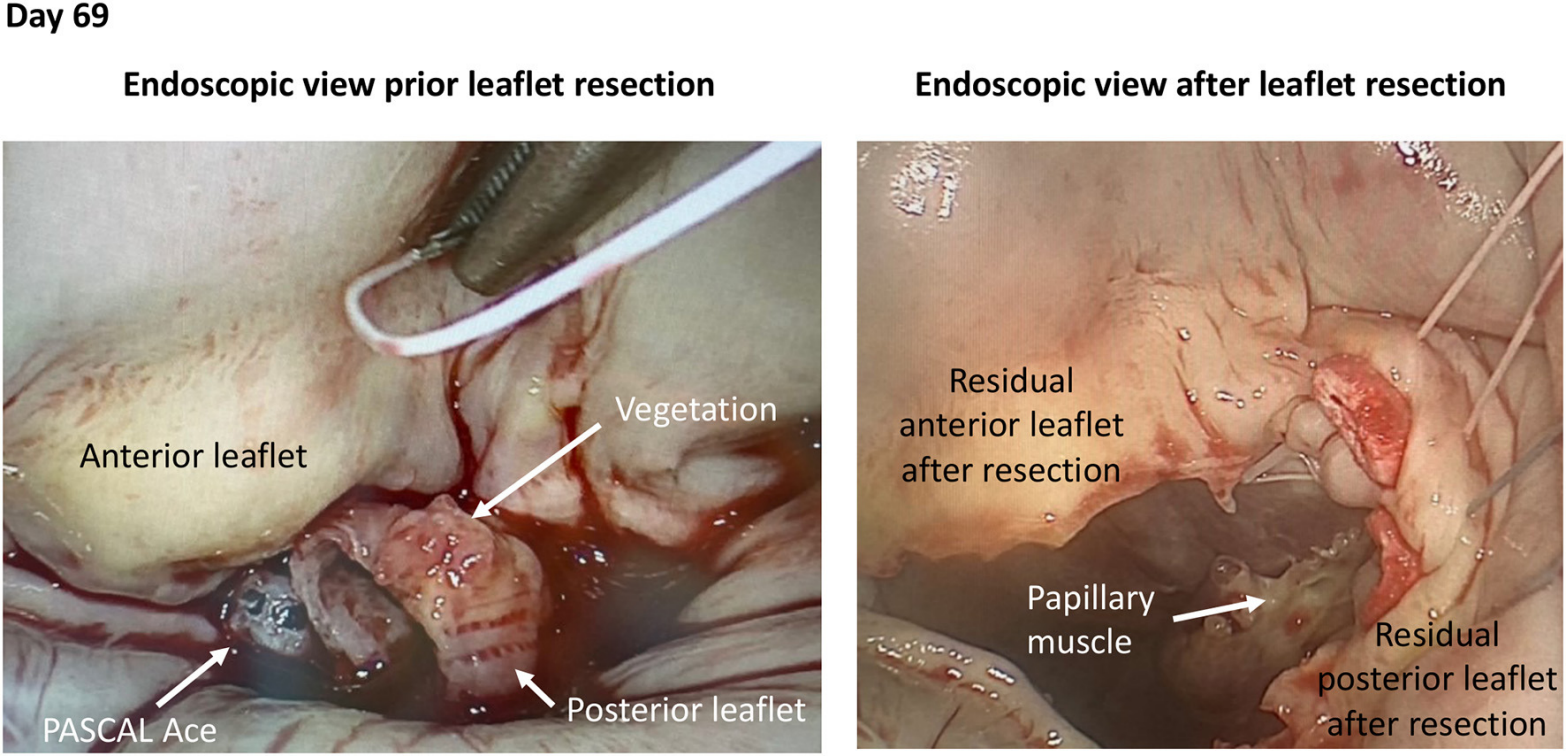
Intraoperative views of PASCAL Ace infective endocarditis. Intraoperative pictures during endoscopic mitral valve repair in infective endocarditis of a PASCAL ACE device.

The antibiotic regimes with Ampicillin were continued and transesophageal echocardiography (TOE) was scheduled. In TOE, IE of the PASCAL Ace device was confirmed by a vegetation accompanied by a mild to moderate mitral regurgitation (Figure 2, Day 36; Supplementary Videos 3, 4). Patient was transferred to the Cardiology ward and the local endocarditis team (infectious disease specialist, cardiologist, microbiologist and radiologist) contacted the cardiac surgeon to discuss explantation of the PASCAL Ace device. Risk score evaluation resulted in a very high operative risk (logEuroscore: 37.1%, Euroscore 2: 5.3%, STS score mortality: 5.3%). While the patient was stable at this time and deemed not suitable for cardiac surgery, the endocarditis team made a decision toward a prolonged 6-week antibiotic regime with an antibiotic combination of Ampicillin 2 g qds and Ciprofloxacin 750 mg td. The choice for Ciprofloxacin was based on its high tissue accessibility. Weekly TOEs were performed (Figure 2; Day 43: Supplementary Videos 5, 6; Day 50: Supplementary Videos 7, 8; Day 57: Supplementary Videos 9, 10). Figure 3 shows the inflammatory markers over the clinical course starting at the timepoint of TEER (day 0). Due to posterior leaflet perforation severe mitral regurgitation developed while PASCAL Ace vegetations were significantly reduced by the antibiotic therapy. Therefore, together with the patient and his relatives, the Endocarditis Team made the decision to perform high risk cardiothoracic surgery. On day 69 the patient underwent successful endoscopic mitral valve replacement with a bioprothesis (St Jude Medical Epic 33 mm, Abbott Vascular, Santa Clara, CA, USA; Figure 4). The second day after the operation the patient was free of catecholamines and extubated. The post-operative histology confirmed ulcerative endocarditis, while microbiological culture of the resected tissue was culture negative for bacterial growth. Another four weeks of antibiotic treatment with Ampicillin 2 g qds followed, which was oralized on day 89. The patient was discharged to rehabilitation on day 90. The final echocardiographic result after minimal-invasive mitral valve replacement showed a good post-operative result. Patient was recommended to have lifelong endocarditis prophylaxis.

## Discussion

Device-related IE following TEER is a rare but severe complication. We reported for the first time a case of endocarditis of a PASCAL Ace device, which was colonized by *P. mirabilis*, a bacterium which is well-known to build biofilms and create bacteremia (1). Therefore, IE is not related to a specific TEER device and beside MitraClip (Abbott Vascular, Santa Clara, CA, USA) PASCAL Ace could be affected.

The incidence of IE following TEER in general ranges between 0 and 2.4% (2–4). Furthermore, a data analysis of the Society of Thoracic Surgery about cardiac surgery following TEER, reveals endocarditis to be responsible for cardiac surgery in 4.5% of all cases (5). One might speculate that with increasing TEER procedure volume more cases of IE will appear in clinical practice. To date, no evaluation in a clinical trial or register exists. A systematic review in 2018 revealed 12 cases of IE following TEER in the literature (6). Most of the cases (75%) occurred in the first months, when device endothelization is incomplete (6). The IE led to severe mitral regurgitation in all patients and the majority underwent cardiac surgery with a high mortality of 42% (6). Furthermore, a literature search revealed 17 cases of IE following TEER in 2020 (7). Staphylococcus aureus accounted for 47.1% and *Enterococcus faecalis* for 11.8% of these cases (7). *P. mirabilis* is a member of the *Enterobacteriaceae* family and it rarely causes IE (1). However, its ability to build biofilms and cause bacteremia is associated with a high risk of prosthetic valve endocarditis (25% of all Proteus IE cases) (1). Therefore, bacteremia with Proteus species in patients following TEER should be considered as high risk for IE. TOE in these patients and a prolonged calculated antibiotic regime should be mandatory. In case of suspected IE, the local endocarditis team should coordinate diagnostics and treatment. Furthermore, especially when device endothelization is incomplete (up to 3 months), IE prophylaxis seems to be mandatory following TEER. This regime avoids bacteremia and lower the risk of bacterial device colonization.

In refractory IE with progressing vegetation in TOE or continuous bacteremia despite of a calculated antibiotic regime cardiac surgery should be discussed despite of a high operative risk. Therefore, also endoscopic mitral valve replacement seems to be a feasible option for patients with IE following TEER.

## Conclusion

Infective endocarditis following TEER is rare and occurs most often in the first months after device implantation, when endothelization is incomplete. *P. mirabilis* has a high ability of building biofilms and bacteremia, which poses a risk for colonization of cardiac devices. In refractory IE after TEER mitral valve replacement should be considered regardless of high operative risk.

## Learning Objectives

- Bacteremia of biofilm-builders such as *P. mirabilis* could cause IE, especially in the first months after TEER, when device endothelization is incomplete. Endocarditis prophylaxis should be mandatory in this vulnerable phase after index procedure.
- Treatment of device-related IE should be guided by a local endocarditis team consisting of infectious disease specialist, cardiologist, microbiologist, radiologist and cardiothoracic surgeon.
- IE after TEER is not a specific device problem and beside MitraClip also PASCAL Ace can be affected.
- In persisting IE with progressing vegetations in TOE or continuous bacteremia despite of a calculated antibiotic regime cardiac surgery is a bailout option despite of a high operative risk.
- Minimally invasive endoscopic mitral valve replacement is if feasible one option for patients with IE following TEER.

## Data Availability Statement

The original contributions presented in the study are included in the article/Supplementary Material, further inquiries can be directed to the corresponding author/s.

## Author Contributions

All authors listed have made a substantial, direct, and intellectual contribution to the work and approved it for publication.

## Conflict of Interest

PB was Proctoring and Center of Excellence for Abbot Vascular and Edwards Lifesciences. The remaining authors declare that the research was conducted in the absence of any commercial or financial relationships that could be construed as a potential conflict of interest.

## Publisher's Note

All claims expressed in this article are solely those of the authors and do not necessarily represent those of their affiliated organizations, or those of the publisher, the editors and the reviewers. Any product that may be evaluated in this article, or claim that may be made by its manufacturer, is not guaranteed or endorsed by the publisher.

## Supplementary Material

Supplementary Video 1 Transesophageal echocardiography at day 26 following mitral valve edge-to-edge repair.

Supplementary Video 2 Transesophageal echocardiography with color doppler at day 26 following mitral valve edge-to-edge repair.

Supplementary Video 3 Transesophageal echocardiography at day 36 following mitral valve edge-to-edge repair.

Supplementary Video 4 Transesophageal echocardiography with color doppler at day 36 following mitral valve edge-to-edge repair.

Supplementary Video 5 Transesophageal echocardiography at day 43 following mitral valve edge-to-edge repair.

Supplementary Video 6 Transesophageal echocardiography with color doppler at day 43 following mitral valve edge-to-edge repair.

Supplementary Video 7 Transesophageal echocardiography at day 50 following mitral valve edge-to-edge repair.

Supplementary Video 8 Transesophageal echocardiography with color doppler at day 50 following mitral valve edge-to-edge repair.

Supplementary Video 9 Transesophageal echocardiography at day 57 following mitral valve edge-to-edge repair.

Supplementary Video 10 Transesophageal echocardiography with color doppler at day 57 following mitral valve edge-to-edge repair.
